# Potato Tuberisation Responses to Drought and a Film-Forming Antitranspirant

**DOI:** 10.3390/plants15131971

**Published:** 2026-06-26

**Authors:** Oluwatoyin Favour Olu-Olusegun, Aidan Farrell, James Monaghan, Peter Kettlewell

**Affiliations:** 1 Crop Science Group, Harper Adams University, Newport, Shropshire TF10 8NB, UK; jmonaghan@harper-adams.ac.uk; 2 Department of Life Sciences, The University of the West Indies, St. Augustine Campus, St. Augustine, Trinidad and Tobago; aidan.farrell@uwi.edu

**Keywords:** Vapor Gard, di-1-*p*-menthene, leaf relative water content, tuber set, tuber size distribution

## Abstract

Film-forming antitranspirants may help potatoes tolerate moderate drought, but their effects on early tuberisation and tuber size distribution remain unclear. Two pot experiments were conducted in a polytunnel (late summer) and a glasshouse (winter–spring), with moderate drought imposed during tuber initiation and early bulking, alone (DT) or combined with an antitranspirant (di-1-*p*-menthene; VGDT). Leaf relative water content (RWC), stolon traits, and tuber yield and size distribution were measured. Moderate drought reduced RWC, stolon number, and tuber set, which indicates the sensitivity of early tuber development to water deficit. VGDT increased leaf RWC under drought from 55% to 71% in Experiment 1 and from 62% to 73% in Experiment 2, while the total tuber number under moderate drought increased from 5.2 to 11.7 tubers plant^−1^ in Experiment 1 and from 6.1 to 10.7 tubers plant^−1^ in Experiment 2. VGDT also increased the number of large (≥9 cm) tubers, shifting size distribution towards marketable classes. Although Vapor Gard improved plant water status and tuber number under drought, it did not restore performance to irrigated levels. These findings indicate its value as a complementary tool to mitigate drought-related losses during tuberisation, not a substitute for irrigation.

## 1. Introduction

Potato (*Solanum tuberosum* L.) is among the world’s most drought-sensitive staple crops, with tuberisation recognised as one of the most vulnerable developmental phases to water deficit. Even brief reductions in soil moisture around stolon initiation can sharply reduce stolon number, tuber set and subsequent bulking, with lasting consequences on yield and marketable size [1,2,3]. In processing cultivars such as Russet Burbank, moderate drought not only depresses yield but also increases the incidence of skin disorders and other quality defects [2,4].

Russet Burbank is one of the most widely grown processing cultivars globally, supplying a large share of the frozen and fresh-fried potato market. It is valued for its tuber shape and frying quality but is known to be sensitive to drought and to develop skin and internal defects under water stress, which makes it a relevant model for testing drought-mitigation strategies in potato processing.

Drought around tuber initiation and early bulking typically reduces leaf area and relative water content (RWC), limits carbon partitioning to stolons, and induces changes in root system architecture and osmotic adjustment that favour deeper or thicker roots, reduced fine-root length density and greater solute accumulation in root tissues, thereby restricting water uptake efficiency and tuber growth [2,5]. These responses constrain the window for stolon elongation and tuber initiation and limit the time available for tuber bulking.

Film-forming antitranspirants based on di-1-*p*-menthene (Vapor Gard, VG) form a thin, flexible film over leaves that reduces transpirational water loss while permitting sufficient gas exchange for photosynthesis [6,7]. Research in other crops shows that such coatings can substantially reduce leaf transpiration and, in some cases, moderately influence photosynthetic rates, thereby stabilising plant water status under moderate evaporative demand. However, the magnitude and direction of photosynthetic responses depend on the environment and the species [8,9].

Recent polytunnel studies on potato have shown that VG can improve plant water status and tuber yield under moderate drought, with little or no benefit under severe deficit, and can reduce drought-related defects such as tuber russeting in susceptible varieties [10,11]. Under moderate deficits, traits such as maintenance of leaf RWC, preserving fine feeder-root function, and sustaining stolon growth appear critical for buffering tuber set and size distribution. These traits help coordinate carbon allocation in favour of tuberisation while some soil water remains available [4,5,10]. However, despite growing agronomic interest, the physiological pathway by which VG influences below-ground processes of stolon growth, tuber initiation and tuber bulking in potato remains poorly resolved and requires experiments that connect leaf water status and below-ground development.

Previous polytunnel studies in this series focused on final yield and drought-related defects under contrasting drought intensities, including severe deficits. By contrast, the present pot experiments in a polytunnel and glasshouse target the tuber initiation and early bulking window, quantify maximum rooting depth, and characterise stolon and tuber traits at early bulking, including a combined environment analysis across the two structures. Rather than emphasising final marketable yield, this study focuses on describing how VG influences plant water status, below-ground development and tuber size distribution in the early bulking stage.

Two pot experiments (Exp. 1 and Exp. 2) were, therefore, designed to quantify the physiological effects of VG on plant water status and below-ground development in Russet Burbank under moderate water deficit. Exp. 1 was conducted in a polytunnel and Exp. 2 in a glasshouse; a well-watered irrigated control was included only in Exp. 2. These pot trials in contrasting seasons examined how VG altered leaf RWC, maximum rooting depth and root dry mass, stolon production, tuber set and the formation of large (≥9 cm) tubers relative to unsprayed droughted plants and, in Exp. 2, a well-watered control. By integrating these measurements, this study tests a conceptual model in which film-forming antitranspirant use stabilises leaf water status, sustains carbon assimilation and stolon activity during the tuber initiation window, and thereby enhances tuber set and bulking under moderate, but not severe, water limitation.

In this context, VG provides a useful case study to test whether moderating transpirational water loss at the leaf surface can shift responses away from stress-driven root thickening and towards the maintenance of fine roots, stolon elongation and timely tuber initiation when soil water is limited but not exhausted. We hypothesised that VG would (i) increase leaf RWC under moderate drought, (ii) maintain or enhance functional maximum rooting depth while reducing stress-induced increases in root dry mass, and (iii) extend the effective stolonisation window, resulting in higher tuber set and a greater proportion of large, marketable tubers compared with unsprayed plants exposed to the same water deficit.

### Objectives

Experiments 1 and 2 were undertaken to quantify and interpret the effects of a film-forming antitranspirant (VG) on potato tuberisation under moderate drought. The specific objectives were to: quantify the impact of VG on leaf RWC as an integrative indicator of leaf water status during tuber initiation and early bulking under moderate drought; determine how VG modifies root system traits under moderate drought, focusing on maximum rooting depth and root dry mass as indicators of below-ground allocation and functional water-foraging capacity; and characterise the effects of VG on stolon development, tuber set and the production of large (≥9 cm) tubers under moderate drought and explore potential relationships between antitranspirant-induced changes in leaf water status with below-ground development and tuber bulking.

## 2. Materials and Methods

### 2.1. Site, Pots and Plant Material

Two pot experiments were conducted at Harper Adams University (Shropshire, UK; 52°46′ N, 2°25′ W). Exp. 1 was grown in a polytunnel and planted on 24 July 2024, whereas Exp. 2 was grown in a glasshouse and planted on 24 January 2025. In the glasshouse (Exp. 2), environmental conditions were controlled as described in Section 2.3. Key dates for planting, drought imposition, and VG applications in Exps. 1 and 2 are summarised in Table 1. Both experiments used 20 L plastic pots (32 cm diameter, 26 cm height) each filled with ~12 kg of John Innes No. 2 compost (LBS Worldwide Ltd., Colne, Lancashire, UK), a pre-mixed loam-based medium with peat, coarse sand and base fertiliser. The processing cultivar Russet Burbank, known for susceptibility to jelly end rot (supplied by McCain Ltd., Montrose, Scotland, UK), was used throughout. One certified seed tuber of uniform size was planted per pot at a depth of 6 cm. Pots were arranged on the polytunnel floor and on benches in the glasshouse with about 0.3–0.4 m spacing between pots to avoid canopy overlap and minimise mutual shading. Plants were staked to prevent lodging and additional self-shading. Basal nutrition was supplied as Elixir Gardens Growmore 7–7–7 (Elixir Garden Supplies, Morecambe, UK), a special blend fertiliser (7–7–7 N–P–K, with added micronutrients) [12], applied at 20 g per pot for both experiments at planting. For insect pest control, Hallmark Zeon (Syngenta, Cambridge, UK) was applied at 4 mL in 5 L water (equivalent to 75 mL ha^−1^ in 200 L spray volume) between 30 and 40 DAP at the first sign of aphid presence. This was done in both experiments, in line with label advice to treat at first signs of attack. Full agronomic practices followed those described in [10]. Pot trials were selected to allow precise control of soil water content and antitranspirant application around tuber initiation.

### 2.2. Experimental Design and Treatments

Both experiments used the same treatment structure, but the irrigated control was included only in Exp. 2: moderate drought without antitranspirant (DT; Exps. 1 and 2); moderate drought plus Vapor Gard (VGDT; Exps. 1 and 2); and irrigated control (IRR; Exp. 2 only). VG refers to the product Vapor Gard (di-1-*p*-menthene), and VGDT denotes the drought treatment receiving VG application. Treatments were arranged in a randomised complete block design within each structure, with n = 6 replicate pots per treatment in Exp. 1 and n = 10 replicate pots per treatment in Experiment 2. Plants were grown to tuber initiation under near-optimal water supply before the drought regime was imposed. The slightly lower replication in Exp. 1 (n = 6) reflected space constraints in the polytunnel due to a concurrent previously published experiment, whereas replication was increased in Exp. 2 (n = 10) to improve statistical power; no formal a priori power analysis was conducted.

### 2.3. Environmental Monitoring

To characterise the growing environment, temperature and relative humidity were recorded throughout both experiments. For each experiment, measurements were taken inside the relevant structure (polytunnel for Exp. 1; glasshouse for Exp. 2). A Tinytag Gemini Ultra 2 data logger (Gemini Data Loggers, Chichester, UK) was mounted at an approximate canopy height to reflect the environment around the foliage in each experiment. Air temperature (°C) and relative humidity (%) were logged automatically at hourly intervals from planting to harvest (approximately 12 weeks in Exp. 1 and 14 weeks in Exp. 2). Vapour pressure deficit (VPD, kPa) was calculated from air temperature and relative humidity following FAO-56 formulation [13]:

$$\mathrm{VPD}=e_{s}(T)\times \left(1-\frac{\mathrm{RH}}{100}\right),$$

where $e_{s}(T)$ is the saturation vapour pressure at air temperature T (°C), estimated as

$$e_{s}(T)=0.6108\times exp\left(\frac{17.27T}{T+237.3}\right).$$

**Figure 1:**
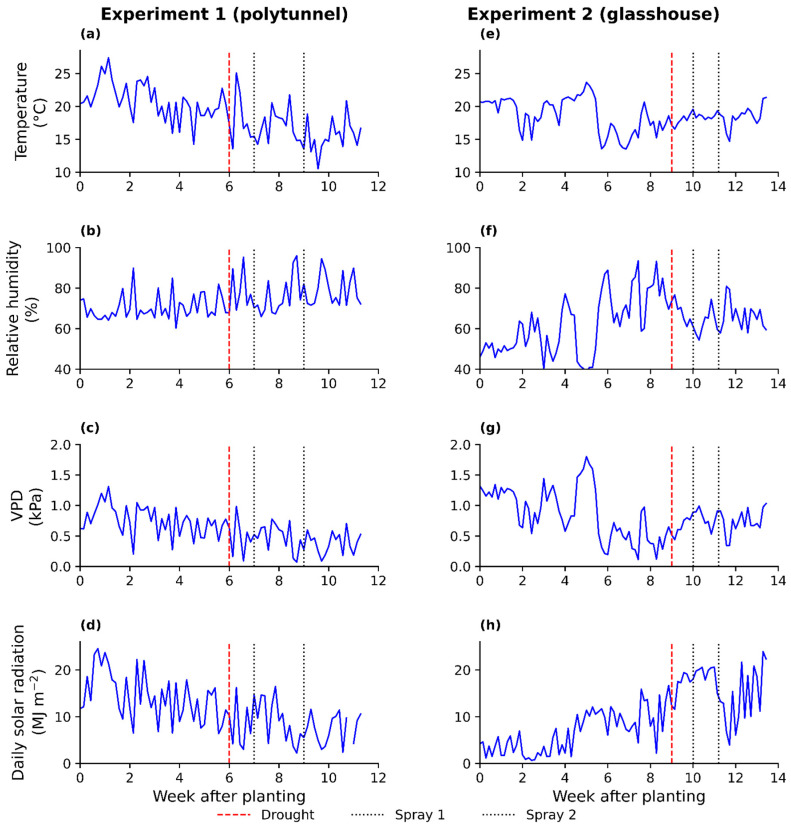
Weekly mean air temperature (°C), relative humidity (%), vapour pressure deficit (VPD, kPa) and daily solar radiation (MJ m^−2^) during Experiment 1 (polytunnel; panels (**a**–**d**)) and Experiment 2 (glasshouse; panels (**e**–**h**)). Each point represents the mean of hourly Tinytag records (temperature and relative humidity) or daily weather-station records (solar radiation) for a given week after planting; lines connect successive weeks to show how environmental conditions changed over the crop cycle. Red dashed vertical lines indicate drought onset; black dotted lines indicate Vapor Gard (Spray 1 and Spray 2).

Daily global solar radiation (MJ m^−2^) for both experiments was obtained from the on-site weather station adjacent to the polytunnel and glasshouse. In Exp. 2, plants additionally received supplementary LED lighting in the glasshouse, where a 16 h photoperiod (16 h light/8 h dark) was provided with lamps switching on automatically when incident light fell below 30 kilolux (~770 µmol m^−2^ s^−1^ PPFD). For graphical presentation and to aid interpretation of seasonal and structural differences between the polytunnel and glasshouse environments, daily values of temperature, relative humidity, VPD and global solar radiation were summarised as weekly means (Figure 1).

### 2.4. Drought Imposition and Volumetric Water Content

Soil water status was monitored throughout both experiments using a portable FieldScout TDR-100 (Spectrum Technologies, Aurora, IL, USA) fitted with 20 cm rods, which were pushed vertically into the substrate of each pot. VWC was recorded every 2–3 days and used to adjust irrigation volumes. Water-release characteristics of the John Innes No. 2 compost gave a field capacity of approximately 45% VWC and an estimated permanent wilting point of about 7.5% VWC [14], corresponding to a plant available water capacity (AWC) of 37.5%. For the moderate drought treatments (DT, VGDT), pots were maintained at a target VWC of approximately 18.8%, corresponding to about 30% of the compost’s available water content. In the IRR control (Exp. 2), pots were maintained near 43% VWC to approximate field capacity.

In Exp. 1, tuber initiation began at 38 days after planting (DAP), and in Exp. 2, it began at 54 DAP, as determined by destructive sampling of 2–3 plants per treatment at 7-day intervals and inspection of underground stems for the first visible tuber initials or swollen stolon tips. Moderate drought (DT, VGDT) was initiated at tuber initiation (38 and 54 DAP in Exps. 1 and 2, respectively), with soil water content progressively reduced thereafter to the target 30% AWC threshold and maintained through the early bulking stage. Irrigation was applied manually after each VWC measurement to return pots to their respective targets (~18.8% VWC for DT and VGDT, ~43% VWC for IRR; Table 2).

### 2.5. Antitranspirant Application

The film-forming antitranspirant, VG (96% di-1-*p*-menthene, Pinolene^®^, Miller Chemicals and Fertiliser, Hanover, PA, USA), was applied to VGDT plants as a fine foliar mist. The commercial label describes VG as forming a flexible, water-resistant film that reduces transpiration and moisture loss when applied to foliage (https://www.millerchemical.com/products/crop-production-aids/vapor-gard; accessed on 20 March 2026). For both experiments, VG was applied at 5 mL L^−1^ in water. Approximately 80–100 mL of spray solution was applied per plant at each application, wetting the foliage to near runoff. Sprays were applied in the late afternoon under sheltered, low-wind conditions and at air temperatures typically between 16 and 22 °C and relative humidity around 60–80% to minimise drift and ensure uniform coverage. The first application was performed approximately one week after drought imposition, when soil VWC in the drought treatments approached the 30% available water content threshold (AWC, Table 2): this corresponded to 54 DAP in Exp. 1 and 64 DAP in Exp. 2. The second application was performed approximately 10–14 days later (64 DAP in Exp. 1 and 78 DAP in Exp. 2). VG was applied using a hand-held sprayer (Hozelock Exel, Arnas, France) operated at approximately 2 bar, with drift-prevention frames around pots to prevent cross-contamination between treatments. DT and IRR plants received no antitranspirant.

### 2.6. Leaf Relative Water Content (RWC)

Leaf RWC was measured as an integrative indicator of plant water status following the method described in [10], with fully expanded leaves from the mid-canopy sampled after the first VG application at 56 DAP (Exp. 1) and 73 DAP (Exp. 2) during tuber initiation. Fresh weight (FW) was recorded immediately after excision. Samples were floated on deionised water in the dark at 4 °C until fully turgid (typically overnight), blotted dry, and weighed to obtain turgid weight (TW). Samples were then oven-dried at 80 °C to a constant mass and weighed for dry weight (DW). RWC was calculated as: RWC = (FW − DW)/(TW − DW) × 100. Mean RWC per plant and per treatment was then derived for analysis.

### 2.7. Maximum Rooting Depth

Maximum rooting depth was assessed during the same destructive harvest used for stolon and tuber measurements at the end of the experiment, corresponding to 79 DAP in Exp. 1 and 94 DAP in Exp. 2. For each sampled plant, the entire root system was carefully excavated from the pot and washed free of compost under gentle running water to minimise loss of fine roots. Roots were then spread out on a flat surface and arranged to minimise overlap.

Following the approach of [15], the main rooting distance was measured as the linear distance from the point of primary root insertion at the stem base to the deepest visible root tip, providing an index of effective rooting depth under the different water and antitranspirant treatments.

### 2.8. Root Dry Mass (RDM)

Washed roots from each plant were blotted on absorbent paper to remove surface moisture, and fresh root mass was recorded using an analytical balance. Root systems were then placed in paper bags and oven-dried at 80 °C for 48 h, or until constant weight, and weighed again to obtain root dry mass (RDM) per plant.

### 2.9. Stolon Development and Tuber Traits

At the final harvest (see Section 2.7), conducted 4 weeks after the onset of tuber initiation and early bulking, the number of stolons and tubers per plant was recorded. Stolons were exposed by carefully removing compost by hand and with gentle washing, and the total stolon number per plant was counted as all underground stems bearing at least one visible tuber or a clearly swollen stolon end indicative of tuber initiation. All tubers attached to the plant were counted to obtain the total number of tubers per plant. Tubers were then measured for maximum length using a ruler; those ≥9 cm were classified as large, reflecting a threshold chosen to represent tubers of a size typically accepted for processing-grade markets in the UK. The number of tubers ≥ 9 cm per plant was used as an index of large, marketable tuber production.

### 2.10. Statistical Analysis

All statistical analyses were carried out using R version 4.4.1 (R Core Team, Vienna, Austria; https://www.r-project.org, accessed in January 2026). Data for Exp. 1 and Exp. 2 were analysed separately because the experiments were conducted in different environments (polytunnel vs. glasshouse) and seasons. For each experiment, treatment effects on leaf RWC, maximum rooting depth, root dry mass, stolon number, number of tubers and number of tubers ≥ 9 cm were evaluated using Welch’s analysis of variance (ANOVA) to account for variance heterogeneity among treatments within a randomised complete block design, with treatment specified as a fixed effect and block as a random effect. Model assumptions of normality and homogeneity of variance were assessed using residual diagnostic plots. Group means are reported in Supplementary Table S1, and pairwise treatment comparisons are reported in Supplementary Table S2. Where overall treatment effects were detected, Games–Howell post hoc tests were used for pairwise comparisons. Experiment 1 involved only one comparison (DT vs. VGDT), whereas Experiment 2 involved three treatment comparisons (IRR vs. DT, IRR vs. VGDT, and VGDT vs. DT).

For the VWC time courses, treatment and DAP effects were assessed using two-way ANOVA on repeated measurements, and simple contrasts between DT and VGDT on each DAP were summarised using Welch’s *t*-tests (Table S3).

To assess whether treatment responses were consistent across environments, a combined analysis was also performed using data from DT and VGDT treatments in both experiments. Combined analyses across environments were conducted using linear mixed models with environment and treatment as fixed factors and block nested within environment; where block variance was negligible, fixed-effects models gave equivalent results.

## 3. Results

### 3.1. Environmental Conditions

In Exp. 1 (polytunnel, 24 July–11 October 2024), the weekly mean air temperature during the crop cycle was generally warm, with daily means ranging from about 11 to 27 °C (overall mean ~19 °C). Relative humidity mostly lay between 60 and 96% (mean ~74%), so VPD remained moderate, typically 0.1–1.3 kPa (mean ~0.6 kPa). Daily global solar radiation was relatively high for a UK summer, with values between 2 and 25 MJ m^−2^ (mean ~11.5 MJ m^−2^), declining during tuber bulking but still providing ample light for canopy and tuber development. Figure 1 summarises the weekly means of temperature, relative humidity, VPD and radiation, with vertical markers indicating drought onset and VG sprays, and a seasonal summary of the minimum, mean, and maximum values for both experiments is provided in Table 3.

In Exp. 2 (glasshouse, 24 January–28 April 2025), the mean air temperatures were slightly cooler overall, ranging from about 14 to 24 °C (mean ~18.6 °C), but relative humidity was more variable (38–93%, mean ~63%) and VPD spanned a wider range (0.1–1.8 kPa, mean ~0.8 kPa). Daily global solar radiation during this winter–spring period averaged slightly lower than in Exp. 1 (mean ~9.8 MJ m^−2^, range 0.7–24 MJ m^−2^), and plants also received supplementary LED lighting in the glasshouse (16 h photoperiod, switching on below 30 kilolux, ~770 µmol m^−2^ s^−1^ PPFD), which increased incident radiation, particularly during early growth. The combination of relatively warm temperatures, lower natural radiation, and an extended photoperiod in Exp. 2 implies a higher temperature: light ratio than in the polytunnel, conditions known to favour stem elongation in greenhouse crops [16,17] and consistent with the taller canopies observed in the glasshouse experiment.

### 3.2. Soil Volumetric Water Content

**Figure 2:**
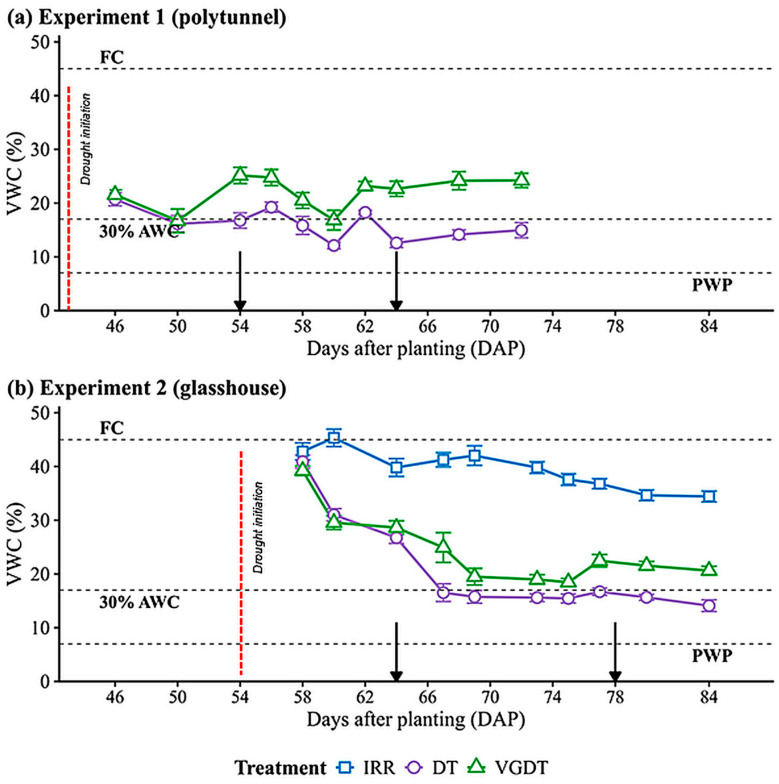
Changes in volumetric soil water content (VWC, %) over time in (**a**) Experiment 1 (polytunnel) and (**b**) Experiment 2 (glasshouse) for potato plants grown under three soil moisture treatments: a fully irrigated control (IRR; Experiment 2 only), drought without an antitranspirant (DT), and drought with the film-forming antitranspirant Vapor Gard (VGDT). Red dashed vertical lines indicate the initiation of drought (dry-down), and vertical arrows indicate the days on which Vapor Gard was applied—54 and 64 DAP in Experiment 1, and 64 and 78 DAP in Experiment 2. The target VWC for the drought treatments (DT and VGDT) was 30% available water capacity (~17% VWC). Horizontal dashed lines mark field capacity (FC ~45% VWC), the 30% AWC threshold (~17% VWC) and the estimated permanent wilting point (PWP ~7% VWC). Symbols represent treatment means (n = 6 per treatment per time point in Experiment 1; n = 10 in Experiment 2), and error bars represent the standard error of the mean. Treatment effects were assessed by two-way ANOVA (Treatment × DAP); the treatment effect was highly significant in both experiments (*p* < 0.001). Abbreviations: FC, field capacity; PWP, permanent wilting point; AWC, available water capacity; IRR, irrigated; DT, drought; VGDT, drought + Vapor Gard; DAP, days after planting; VG, Vapor Gard. Pairwise statistical comparisons (Welch’s *t*-tests) are reported in Supplementary Table S3.

In Exp. 1 (polytunnel), DT and VGDT did not differ at 46 DAP (20.7% vs. 21.6% VWC; *p* = 0.59) or 50 DAP (16.1% vs. 16.7%; *p* = 0.84). After the first VG application (54 DAP), VWC in VGDT increased to 25.2% and thereafter exceeded that of DT by ~4–10 percentage points; following the second application (64 DAP), VGDT averaged 23–24% VWC, while DT remained at 12–15%. Treatment, DAP and their interaction were all significant (*p* < 0.001, *p* < 0.001 and *p* < 0.01, respectively; Figure 2a; Table S3). This indicates that VGDT not only improved plant water status but also conserved soil moisture, with VG-treated pots consistently wetter than untreated droughted pots by approximately 4–10 percentage points.

In Exp. 2 (glasshouse), IRR, DT and VGDT were similar at 58 DAP (42.8%, 40.9% and 39.2% VWC; *p* = 0.15). IRR declined gradually from 45.3% to 34.4% VWC between 60 and 84 DAP. After drought imposition at 62 DAP, DT fell from 31.0% to 16.5% VWC (60–67 DAP), whereas VGDT declined from 29.6% to 24.9%. Thereafter, VGDT remained 3–8 percentage points wetter than DT, with DT stabilising at 14–17% VWC. Treatment, DAP and their interaction were all highly significant (*p* < 0.001; Figure 2b; Table S3). At the time of the first VG application, the DT pots in Exp. 1 were already close to the 30% AWC threshold, whereas in Exp. 2, soil VWC was still higher when VG was first applied (Figure 2), which indicates that the plants in Exp. 1 experienced a more severe deficit by the time VG was introduced.

### 3.3. Leaf Water Status (RWC)

Moderate drought substantially reduced leaf RWC, and VG consistently mitigated this effect in both experiments. In Exp. 1, RWC under drought without antitranspirant (DT) averaged 54.9%, whereas VGDT plants reached 71.1%, a difference that was statistically significant (*p* = 0.011). In Exp. 2, irrigated plants (IRR) had the highest RWC (86.9%), DT plants declined to 62.4%, and VGDT plants were intermediate at 72.6%. IRR differed significantly from both DT (*p* < 0.001) and VGDT (*p* = 0.0020), whereas VGDT also had significantly higher RWC than DT (*p* = 0.022) in the same direction as in Exp. 1. Thus, VG alleviated but did not eliminate the reduction in RWC caused by moderate drought.

Although VG consistently increased RWC under moderate drought in both experiments, the magnitude of this effect varied among experiments. Because the experiments spanned a range of evaporative demand, we examined whether variation in VG’s relative effectiveness was associated with differences in vapour pressure deficit (VPD) across experiments.

### 3.4. Root System Traits

In Exp. 1, maximum rooting depth did not differ significantly between DT (37.8 cm) and VGDT (42.5 cm; *p* = 0.38). In Exp. 2, however, droughted plants without VG had the deepest roots (55.2 cm), while VGDT plants had intermediate length (45.3 cm) and IRR the shortest roots (36.2 cm; overall treatment effect *p* < 0.001). DT had significantly longer roots than IRR (*p* < 0.001) and VGDT (*p* < 0.001), and IRR also differed significantly from VGDT (*p* = 0.027).

**Figure 3:**
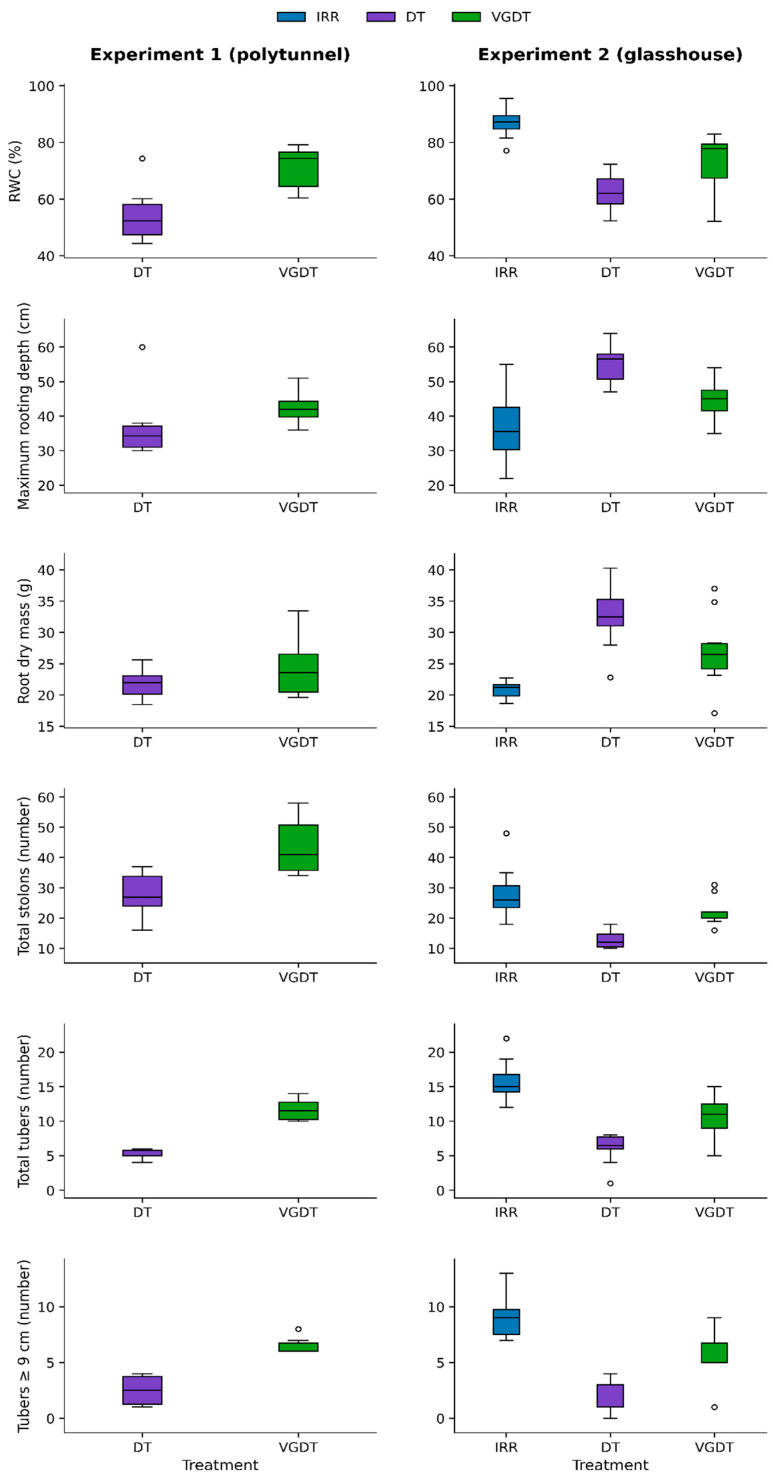
Boxplots showing the effects of drought and antitranspirant treatment (VGDT) on tuberisation-related traits in *Solanum tuberosum* L. cv. Russet Burbank grown under Experiment 1 (polytunnel; treatments: DT and VGDT) and Experiment 2 (glasshouse; treatments: IRR, DT, VGDT) conditions. Traits are presented from top to bottom as: leaf relative water content (RWC, %), maximum rooting depth (cm), root dry mass (g), total stolon number, total tuber number, and number of tubers ≥ 9 cm. Boxes represent the interquartile range (IQR), horizontal lines denote medians, whiskers extend to 1.5 × IQR, and circles indicate outliers. Treatment colours are IRR (blue), DT (purple), and VGDT (green). Group means (± SD, n) for all traits are reported in Supplementary Table S1, and pairwise statistical comparisons (Games–Howell post hoc tests following Welch’s ANOVA) are reported in Supplementary Table S2.

Root dry mass (RDM) showed no significant treatment effect in Exp. 1 (DT 21.8 g, VGDT 24.5 g; *p* = 0.30). In Exp. 2, RDM was lowest in IRR (20.9 g), highest in DT (32.4 g) and intermediate in VGDT (27.2 g; overall treatment effect *p* < 0.001). DT had significantly higher RDM than IRR (*p* < 0.001), and VGDT also had higher RDM than IRR (*p* = 0.008), and DT had significantly higher RDM than VGDT (*p* = 0.032). Thus, treatment effects on RDM were detected only in Exp. 2 (Figure 3).

### 3.5. Stolon Development and Tuber Set

VG increased stolon development and tuber set (total tuber number) under moderate drought in both experiments. In Exp. 1, VGDT increased total stolons from 27.7 to 43.7 per plant (*p* = 0.012). In Exp. 2, total stolons were greatest in IRR (29.1), strongly reduced in DT (12.5) and partly restored in VGDT (21.7; overall treatment effect *p* < 0.001), with VGDT significantly higher than DT (*p* < 0.001) and IRR also higher than VGDT (*p* = 0.046). These stolon responses translated into a higher tuber set. In Exp. 1, VGDT more than doubled the total tuber number compared to DT (11.7 vs. 5.2 tubers plant^−1^; *p* < 0.001). In Exp. 2, tuber number showed the same pattern across treatment as stolon number (IRR 15.8 > VGDT 10.8 > DT 6.1 tubers plant^−1^; overall treatment effect *p* < 0.001), and all pairwise contrasts were significant (*p* ≤ 0.001).

### 3.6. Tuber Bulking (≥ 9 cm Tubers)

VG also improved the production of large tubers under moderate drought. In Exp. 1, VGDT increased the number of tubers ≥ 9 cm from 2.5 to 6.5 per plant (*p* < 0.001). In Exp. 2, IRR produced the highest number of tubers (9.1 per plant), DT produced very few (1.9), and VGDT partially restored this class to 5.5 per plant (overall treatment effect *p* < 0.001); VGDT had significantly more large tubers than DT (*p* < 0.001) and significantly fewer than IRR (*p* < 0.001). Expressed as proportions, in Exp. 2, drought without VG shifted the crop towards smaller tubers (<9 cm), whereas VGDT partly reversed this shift. Under VGDT, the proportion of large tubers (≥9 cm) was substantially higher than under DT but remained lower than that under IRR (*p* < 0.001 for DT vs. VGDT and IRR vs. VGDT).

To formally test whether the response to VG under moderate drought differed between environments, the data from the polytunnel and glasshouse experiments were combined for the DT and VGDT treatments only, with environment and treatment as fixed factors and block nested within environment. No significant Environment × Treatment interaction was detected for relative water content (RWC; *p* = 0.35), total stolon number (*p* = 0.13), or the number of large tubers (≥9 cm; *p* = 0.69). By contrast, VG significantly increased RWC, stolon number and large tuber number across environments. These results indicate that while absolute trait values differed between environments, the magnitude of the VG effect under moderate drought was consistent across experimental contexts.

## 4. Discussion

The two pot experiments provide a framework to examine how the film-forming antitranspirant Vapor Gard (VG) modifies potato responses to moderate drought. In both environments, plants with VG showed a higher RWC, greater stolon activity and a higher tuber set than untreated droughted plants, with stronger responses in the glasshouse than in the polytunnel. In the following sections, these patterns are interpreted in relation to proposed mechanisms of antitranspirant action, the differences in VPD and drought patterns between experiments (Figure 1 and Figure 2; Table S3), and published work on potato drought physiology.

### 4.1. Antitranspirant Effects on Plant Water Status

Moderate drought reduced leaf RWC markedly, and VG consistently increased RWC relative to untreated droughted plants in both seasons, which indicates improved leaf water status under water limitation. In Exp. 1, VG raised mean leaf RWC from 55% under moderate drought to 71%, while in Exp. 2, it increased RWC from 62% to 73%, which represents a roughly 20–30% higher RWC than that in the untreated droughted plants. This pattern is consistent with our recent polytunnel work, which showed that VG improves leaf water status and yield under moderate drought in potatoes [10,11]. Similar responses have been reported in other crops, where VG reduced transpiration and improved plant water relations, although the magnitude of benefit varies with environment and genotype [7]. The fact that RWC under VGDT in the present study remained below irrigated levels supports the view that film-forming antitranspirants provide partial water saving rather than full physiological protection, especially when evaporative demand is high [11]

Although the RWC responses to VG appeared greater in the glasshouse than in the polytunnel, the mean VPD was only moderately higher in the glasshouse (0.8 vs. 0.6 kPa). This indicates that differences in evaporative demand alone cannot fully explain the stronger VG effect observed in that experiment. At the higher VPD observed in Exp. 2, VG still produced a positive but relatively small increase in RWC, which indicates that some water-status benefit can occur even under high evaporative demand, although its proportional impact is reduced compared to that in experiments conducted at lower VPD. In addition, the VWC trajectories show that the DT plants in Exp. 1 had already had a lower soil water content when VG was first applied than the DT plants in Exp. 2 (Figure 2; Table S3), which suggests that VG was introduced under a more severe water deficit in Exp. 1.

This pattern is consistent with the mode of action of film-forming antitranspirants, which impose a relatively fixed increase in leaf diffusive resistance. At low-to-moderate VPD, this reduction in transpirational flux can substantially improve plant water status. At higher VPD, however, the much stronger atmospheric driving force for water loss reduces the proportional effectiveness of the treatment. Experimental and modelling studies similarly indicate that partial restrictions on transpiration can stabilise leaf water status under moderate evaporative demand but become less effective as VPD increases [9,18].

Taken together with the VWC data (Figure 2; Table S3), these results suggest that between-experiment differences in VG effectiveness primarily reflect the pattern and severity of soil water deficit over time, particularly soil moisture status at the time of antitranspirant application. If VG effectiveness depends at least in part on evaporative demand and the magnitude of water-status improvement, downstream traits such as root allocation and tuberisation might be expected to scale with the VG effect on RWC rather than with treatment alone.

### 4.2. Root Responses and Below-Ground Allocation

Consistent with the VPD-dependent effects of VG on leaf water status, root growth and below-ground allocation differed between treatments and experiments, which indicates that changes in leaf water status influenced the balance between root growth and tuber development. In Exp. 2 (glasshouse), moderate drought increased root dry mass, and DT also produced the longest roots, which indicates a typical stress-induced shift towards below-ground biomass investment and deeper soil exploration. This is consistent with reports that some potato genotypes respond to water deficit by increasing root depth or root mass, although the direction and magnitude of response vary among cultivars and experiments [1,2,5]. Under VGDT, maximum rooting depth remained high while root dry mass was moderated relative to DT, which may indicate a more favourable pattern of below-ground allocation. However, because total root length and root diameter were not measured, any inference about root hydraulic efficiency or fine-root proliferation should be considered tentative. Such a pattern is consistent with recent syntheses suggesting that drought-tolerant potatoes often maintain or increase maximum rooting depth while limiting excessive carbon allocation to coarse roots, thereby supporting water uptake without unduly penalising tuber growth [2,19]. In Exp. 1 (polytunnel), VG had little effect on root traits, which reinforces the conclusion that antitranspirant impacts on root systems are context-dependent and strongest when VG produces a clear improvement in leaf water status relative to untreated drought.

### 4.3. Stolon Development and Tuber Set Under Moderate Drought

Drought strongly reduced stolon numbers and total tuber set in the moderate-drought treatment compared to irrigation, which confirms that tuberisation is among the most drought-sensitive phases of potato development [4,5]. In Exp. 2 (glasshouse), the application of VG under moderate drought substantially increased total stolons (from 12.5 to 21.7 stolons plant^−1^) and almost doubled tuber number (from 6.1 to 10.8 tubers plant^−1^) relative to untreated drought, in parallel with a partial recovery of leaf RWC, a response we have observed previously [11]. This suggests that improved plant water status was sufficient to maintain stolon activity and tuber initiation despite reduced soil moisture. Because stolons were counted only when they bore a visible tuber or swollen tip, purely vegetative stolons without visible initiation were not included. The total stolon number may be slightly underestimated; however, the measure directly reflects the stolons participating in tuber initiation.

The polytunnel and glasshouse experiments show that VG can support stolon activity and tuber set under moderate water deficit across contrasting environments, but the effect size differs between seasons. In the glasshouse experiment (Exp. 2), conducted with a fully irrigated reference and a sustained moderate soil water deficit in DT and VGDT, VG produced clear gains in RWC, stolon number and tuber set compared with DT. By contrast, in the polytunnel experiment (Exp. 1), where VWC was already lower at the time of VG application, VG’s effects on yield components were smaller and, in some cases, not statistically significant. Across our experiments and previous work, VG tended to be most effective when applied under moderate rather than very severe soil water deficit, consistent with the idea that some available water is required for antitranspirant-mediated water saving to translate into improved tuberisation.

This pattern mirrors previous findings in wheat, which also showed that soil moisture deficit at the time of antitranspirant application was a key determinant of yield response [20]. These results support the conclusion that differences in VG effectiveness among experiments are likely influenced by drought severity and the timing of antitranspirant application. This suggests that the magnitude and detectability of VG benefits for tuber set depend on the environmental context, particularly on the pattern and severity of soil water deficit over time, rather than on irrigation treatment alone. Although potatoes are rarely grown in glasshouses commercially, the combination of a semi-controlled polytunnel environment (Exp. 1) and a glasshouse with a full-irrigation reference (Exp. 2) provides complementary evidence that VG can buffer tuber set against moderate water deficits, while also highlighting the need for further validation under fully field-grown conditions.

Earlier field and pot studies have shown that water stress can either enhance or reduce stolon number, depending on the stress timing and genotype (e.g., deeper or more extensive root systems can contribute to tolerance), while total stolon length and tuber yield are usually depressed by drought [4,5]. The present results extend this understanding to show that a film-forming antitranspirant can partly decouple tuber set from moderate drought by stabilising leaf water relations (higher RWC in VG-treated plants), in line with the observed differences in soil water status between DT and VGDT.

### 4.4. Effects on Bulking and Tuber Size Distribution

Across both experiments, VG increased not only the total number of tubers but also the number of large (≥9 cm) tubers under moderate drought, shifting the size distribution towards the marketable class at the early bulking harvest [11]. In this study, tubers ≥ 9 cm were defined as marketable-size tubers (Section 2.9), so the VG-induced increase in this class directly reflects an improved marketable yield under moderate drought. This pattern is consistent with many reports that drought during tuber initiation and bulking reduces both tuber number and size and can severely compromise marketable yield and quality when soil moisture falls below the optimal range for potato growth [2,4,10,21]. However, other studies have shown that under moderate water deficits, some varieties experience large reductions in total yield but relatively stable tuber size distributions, implying that tuber number is more affected than size in those cases and underscoring that bulking responses to drought are strongly genotype- and environment-dependent [2,22]. Several studies emphasise that water availability during bulking is typically a dominant driver of final tuber size, with quantitative trait studies and multi-environment trials showing strong effects of in-season evapotranspiration or soil water status on the proportion of large tubers; even moderate deficits can reduce the proportion of large tubers in stress-sensitive cultivars [2,21,22]. By maintaining higher RWC and sustaining stolon and tuber development through early bulking, VG appears to have preserved tuber development under drought in the present study, thereby limiting the shift towards small tubers that is typically observed under water stress [4,11]. A plausible mechanism is that VG prolongs the effective tuber initiation window and supports continued stolon activity under moderate drought, which allows a greater fraction of initiated tubers to continue bulking into the ≥9 cm marketable size class.

### 4.5. Environmental Context and Limitations

The experiments were conducted in pots, where root confinement and pot-edge effects can exacerbate drought responses compared with field soils, so field validation is required before broad recommendations can be made. Although this study quantified key structural traits, it did not measure gas exchange, photosynthetic rate, detailed carbon partitioning, or hormone dynamics (e.g., ABA, GA, cytokinins), nor did it quantify shoot biomass or non-structural carbohydrate pools, which limits mechanistic inference about the regulatory pathways linking VG application to stomatal behaviour, assimilate supply and tuber bulking. These aspects are, therefore, discussed qualitatively in relation to leaf RWC and tuber traits, and the proposed physiological mechanisms are framed as hypotheses to be tested in future work. In the early stages of Exp. 2, some plants also showed mild leaf oedema, which later disappeared as the canopy developed and environmental conditions changed over time; because this symptom resolved across all treatments, it is unlikely to have driven the differential effects of VG but does underline the sensitivity of the system to microclimate. These environmental differences likely contributed to the clearer VG effects observed in the glasshouse experiment (Exp. 2) compared to the more variable polytunnel conditions in Exp. 1, where higher VPD and the consistent, albeit modest, VWC advantage of VGDT over DT after VG application (Figure 2; Table S3) influenced the size of VG responses. A single processing cultivar, Russet Burbank, was tested; responses in other cultivars, particularly those with contrasting root systems or drought tolerance profiles, may differ. The irrigated control was included only in Exp. 2, which limits the direct comparison of VG effects under drought with fully irrigated performance in the polytunnel experiment; this is noted when interpreting environment-specific responses. Finally, the moderate sample sizes (n = 6 in Exp. 1 and n = 10 in Exp. 2) reflect the space and resource constraints inherent to root and stolon excavations in pot systems and may limit the power to detect smaller treatment effects, particularly for root traits; this is acknowledged in the interpretation of the marginal results and motivates future work with larger sample sizes.

### 4.6. Physiological Interpretation and Implications

These experiments support a physiological interpretation in which VG reduces transpirational water loss, leading to higher leaf RWC, moderated root thickening, sustained stolon development and increased tuber initiation under moderate drought. This pattern is consistent with the idea that stabilising leaf water status can help maintain carbon supply to stolons and developing tubers even when soil moisture is limited. Exploratory Pearson correlations across the DT and VGDT treatments were consistent with the proposed mechanism: plants with higher leaf RWC and more stolons tended to produce more large tubers, whereas associations involving soil VWC and maximum rooting depth were weak (Table S4). A weak, marginally significant correlation between leaf RWC and soil VWC suggests that VG-related differences in soil moisture contributed to, but did not fully determine, variation in plant water status. Together with the higher soil VWC under VGDT than under DT, these results support a dual mechanism in which VG both reduces canopy transpiration and conserves soil water, thereby improving plant water status under moderate drought. These patterns suggest that VG reduced canopy water loss sufficiently to slow soil drying. As a result, VG-treated pots retained higher VWC during the tuber initiation and early bulking window. The taller stems and more expanded canopy in the glasshouse would be expected to increase whole-plant transpiration demand, as transpiration generally scales with leaf area and canopy size [23], which is consistent with the greater VG-related improvements in water status and tuberisation observed in that environment.

This aligns with the wider understanding that improved plant water status under drought can stabilise photosynthesis and carbon partitioning, thereby protecting tuberisation and bulking [2,4,21]. In many crops, drought induces ABA-mediated stomatal closure, reducing transpiration. This response also constrains carbon assimilation. Strategies that moderate water loss without excessively limiting stomatal conductance can, therefore, help sustain source strength under water deficit [24,25,26]. In potatoes, water stress alters assimilate supply and tuber partitioning. Reduced carbon flow to developing stolons and tubers contributes to lower tuber set and size [1,27]. The present results are consistent with VG helping to maintain leaf water status at levels that support stolon activity and tuber bulking. However, the underlying hormonal and photosynthetic responses were not measured and remain to be characterised.

In line with this mechanism, field evidence from cereals further shows that yield improvement with VG is driven primarily by reduced transpiration, with reductions in endogenous ABA playing a secondary role [8]. Previous work has also evaluated metabolic antitranspirants in potato, which reduce transpiration by directly affecting stomatal metabolism or associated photosynthetic processes. Compared with such compounds, film-forming antitranspirants like VG primarily act as physical diffusion barriers at the leaf surface. The barrier reduces transpirational water loss, thereby helping to maintain plant water status and reduce tuber physiological disorders under moderate drought.

These findings indicate that VG provides partial water saving. This may be sufficient to buffer key developmental stages such as stolon formation and early bulking under moderate drought. However, it may be insufficient to fully restore irrigated performance. This is consistent with the observation that droughted plants treated with VG did not reach the water status or yield of irrigated controls, reinforcing the need to consider antitranspirants as complementary management tools rather than a substitute for adequate irrigation, particularly under severe or prolonged drought.

## 5. Conclusions

Vapor Gard enhanced leaf water status, stolon development, tuber initiation and the production of large (≥9 cm), marketable-size tubers under moderate drought, but plants treated with Vapor Gard did not reach the performance of fully irrigated controls. Its benefits were greatest when soil moisture was moderately, rather than severely, depleted. Overall, Vapor Gard offers a useful complementary option to reduce drought-related losses in potato during tuber initiation and early bulking, but it should be used alongside, not instead of, appropriate irrigation management.

## Figures and Tables

**Table 1:** Summary of developmental stages, drought initiation and antitranspirant application timing in Experiments 1 and 2.

Experiment 1	Experiment 2					
Week	Week	Stage	DAP (Days After Planting) at Drought Initiation	% of AWC	AT	Assessments
1–2	1–4	Establishment		100%	-	-
3–5	5–8	Stolon initiation		100%	-	-
6–9	9–12	Tuber initiation	38 (Exp. 1) 54 (Exp. 2)	Dry-down	-	Porometer
7 (13 September)	10 (27 March)	Tuber initiation	-	30%	Spray 1 (54 DAP Exp. 1; 64 DAP Exp. 2)	Porometer
8	11	Tuber initiation	-	30%	-	Porometer/ RWC
9	12	Tuber initiation	-	30%	Spray 2 (64 DAP Exp. 1; 78 DAP Exp. 2)	Porometer
10–12	13–14	Tuber filling		30%	-	Harvest

**Table 2:** Soil moisture measurement thresholds for John Innes No. 2 compost, showing field capacity (FC), permanent wilting point (PWP), available water content (AWC), and irrigation regimes for well-watered (Experiment 2 only) and drought treatments. All percentages are volumetric water content (VWC) expressed as a percentage of soil volume.

Parameter	Soil moisture (% VWC)	Notes
Field Capacity	45% VWC	
Wilting Point	7.5% VWC	
AWC	37.5% VWC	=FC − PWP
Well-watered	42.75% VWC (95% FC)	IRR (Exp. 2)
DT threshold	18.75% VWC (30% AWC)	Moderate drought
VGDT threshold	18.75% VWC (30% AWC)	Moderate drought

**Table 3:** Seasonal summary of daily air temperature (°C), relative humidity (%), vapour pressure deficit (VPD, kPa) and daily solar radiation (MJ m^−2^) during Experiment 1 (24 July–11 October 2024) and Experiment 2 (24 January–28 April 2025). Reported values are the minimum, maximum, and mean calculated from daily observations.

Experiment	Condition	Min	Max	Mean
Exp. 1	Temperature (°C)	10.5	27.4	18.8
Exp. 1	Relative humidity (%)	60.3	95.9	73.8
Exp. 1	VPD (kPa)	0.1	1.3	0.6
Exp. 1	Daily solar radiation (MJ m^−2^)	2.2	24.5	11.5
Exp. 2	Temperature (°C)	13.5	23.7	18.6
Exp. 2	Relative humidity (%)	38.4	93.4	63.1
Exp. 2	VPD (kPa)	0.1	1.8	0.8
Exp. 2	Daily solar radiation (MJ m^−2^)	0.7	23.9	9.7

## Data Availability

The original contributions of this study are included in the article. Further inquiries may be directed to the corresponding authors.

## Supplementary Materials

The following supporting information can be downloaded at: https://www.mdpi.com/article/10.3390/plants15131971/s1.
