# Supplementary material for: Potato Tuberisation Responses to Drought and a Film-Forming Antitranspirant
Source: Plants (Basel). 2026 Jun 26;15(13):1971. doi: 10.3390/plants15131971 (PMC13364137; doi:10.3390/plants15131971)
Supplement: Supplementary file 1 [file plants-15-01971-s001.zip › plants-4372977-supplementary.pdf]

Supplementary material

Table S1. Group means (± SD) and sample sizes (n) for all measured variables at the early bulking harvest in Experiment 1 (polytunnel) and Experiment 2 (glasshouse). Variables include leaf relative water content (RWC, %), maximum rooting depth (cm), root dry mass (g), total stolons (number), total tubers (number), and tubers ≥ 9 cm (number). Treatments were irrigated control (IRR; Experiment 2 only), moderate drought (DT), and moderate drought plus Vapor Gard (VGDT).

**Experiment**	**Treatment**	**n**	**RWC (%)**	**Maximum rooting depth (cm)**	**Root dry mass (g)**	**Stolons (number)**	**Total tubers (number)**	**Tubers ≥ 9 cm (number)**
Exp. 1 (polytunnel)	DT	6	54.9 ± 11.1	37.8 ± 11.3	21.8 ± 2.6	27.7 ± 7.9	5.2 ± 0.8	2.5 ± 1.4
	VGDT	6	71.1 ± 8.2	42.5 ± 5.2	24.5 ± 5.3	43.7 ± 9.9	11.7 ± 1.6	6.5 ± 0.8
Exp. 2 (glasshouse)	IRR	10	87.1 ± 5.5	36.4 ± 9.7	20.9 ± 1.4	28.5 ± 8.4	15.9 ± 2.8	9.2 ± 2.0
	DT	10	62.3 ± 6.8	55.1 ± 5.5	32.7 ± 5.1	12.9 ± 2.8	6.1 ± 2.2	1.8 ± 1.3
	VGDT	10	72.5 ± 10.7	45.1 ± 5.7	27.0 ± 5.7	21.9 ± 4.6	10.7 ± 2.9	5.5 ± 2.1

Table S2. Pairwise statistical comparisons for all measured variables at the early bulking harvest in Experiment 1 (polytunnel) and Experiment 2 (glasshouse). Comparisons were made within each experiment using Welch's analysis of variance (ANOVA) to account for variance heterogeneity. Where overall treatment effects were detected, Games–Howell post-hoc tests were used for pairwise comparisons. Experiment 1 includes the single contrast DT vs VGDT, while Experiment 2 includes IRR vs DT, IRR vs VGDT, and VGDT vs DT. Variables: leaf relative water content (RWC, %), maximum rooting depth (cm), root dry mass (g), total stolons (number), total tubers (number), and tubers ≥ 9 cm (number). *p*-values are rounded to four decimal places; values shown as 0.0000 indicate $p < 0.00005$.

**Experiment**	**Parameter**	**Pair**	***p* Values (Games–Howell)**
Exp. 1 (polytunnel)	RWC (%)	VGDT vs DT	0.0176
Exp. 1 (polytunnel)	Maximum rooting depth (cm)	VGDT vs DT	0.382
Exp. 1 (polytunnel)	Root dry mass (g)	VGDT vs DT	0.3001
Exp. 1 (polytunnel)	Total stolons (number)	VGDT vs DT	0.0122
Exp. 1 (polytunnel)	Total tubers (number)	VGDT vs DT	0.0000
Exp. 1 (polytunnel)	Tubers ≥9 cm (number)	VGDT vs DT	0.0003
Exp. 2 (glasshouse)	RWC (%)	IRR vs VGDT	0.0019
Exp. 2 (glasshouse)	RWC (%)	IRR vs DT	0.0000
Exp. 2 (glasshouse)	RWC (%)	VGDT vs DT	0.0221
Exp. 2 (glasshouse)	Maximum rooting depth (cm)	IRR vs VGDT	0.0274
Exp. 2 (glasshouse)	Maximum rooting depth (cm)	IRR vs DT	0.0001
Exp. 2 (glasshouse)	Maximum rooting depth (cm)	VGDT vs DT	0.0009
Exp. 2 (glasshouse)	Root dry mass (g)	IRR vs VGDT	0.0079
Exp. 2 (glasshouse)	Root dry mass (g)	IRR vs DT	0.0000
Exp. 2 (glasshouse)	Root dry mass (g)	VGDT vs DT	0.0316
Exp. 2 (glasshouse)	Total stolons (number)	IRR vs VGDT	0.0462
Exp. 2 (glasshouse)	Total stolons (number)	IRR vs DT	0.0002
Exp. 2 (glasshouse)	Total stolons (number)	VGDT vs DT	0.0001
Exp. 2 (glasshouse)	Total tubers (number)	IRR vs VGDT	0.0008
Exp. 2 (glasshouse)	Total tubers (number)	IRR vs DT	0.0000
Exp. 2 (glasshouse)	Total tubers (number)	VGDT vs DT	0.0010
Exp. 2 (glasshouse)	Tubers ≥9 cm (number)	IRR vs VGDT	0.0008
Exp. 2 (glasshouse)	Tubers ≥9 cm (number)	IRR vs DT	0.0000
Exp. 2 (glasshouse)	Tubers ≥9 cm (number)	VGDT vs DT	0.0002

Table S3. Contrasts of volumetric water content (VWC) between deficit treatment (DT) and Vapor Gard–deficit treatment (VGDT) across days after planting (DAP). Exp. 1 was conducted under polytunnel conditions and Exp. 2 under glasshouse conditions. Because comparisons involved only two treatments at each individual time point, *p*-values were obtained using Welch's t-tests.

**Experiment**	**DAP**	**Contrast**	***p* Values**
Exp. 1 (polytunnel)	46	DT vs VGDT	0.592
Exp. 1 (polytunnel)	50	DT vs VGDT	0.842
Exp. 1 (polytunnel)	54	DT vs VGDT	0.002
Exp. 1 (polytunnel)	56	DT vs VGDT	0.013
Exp. 1 (polytunnel)	58	DT vs VGDT	0.061
Exp. 1 (polytunnel)	60	DT vs VGDT	0.05
Exp. 1 (polytunnel)	62	DT vs VGDT	0.001
Exp. 1 (polytunnel)	64	DT vs VGDT	<0.001
Exp. 1 (polytunnel)	68	DT vs VGDT	<0.001
Exp. 1 (polytunnel)	72	DT vs VGDT	<0.001
Exp. 2 (glasshouse)	58	DT vs VGDT	0.284
Exp. 2 (glasshouse)	60	DT vs VGDT	0.406
Exp. 2 (glasshouse)	64	DT vs VGDT	0.264
Exp. 2 (glasshouse)	67	DT vs VGDT	0.02
Exp. 2 (glasshouse)	69	DT vs VGDT	0.07
Exp. 2 (glasshouse)	73	DT vs VGDT	0.009
Exp. 2 (glasshouse)	75	DT vs VGDT	0.013
Exp. 2 (glasshouse)	77	DT vs VGDT	<0.001
Exp. 2 (glasshouse)	80	DT vs VGDT	<0.001
Exp. 2 (glasshouse)	84	DT vs VGDT	<0.001

Table S4. Pearson correlation coefficients (r) among soil volumetric water content (VWC), leaf relative water content (RWC), total stolon number, maximum rooting depth and large tuber number (≥ 9 cm) across DT and VGDT treatments. Soil VWC was averaged per plant over the Vapor Gard application period (56–64 DAP in Experiment 1; 67–77 DAP in Experiment 2) to provide a single representative soil-water metric for each plant. Soil VWC showed weak and non-significant correlations with total stolon number, large tuber number and maximum rooting depth ($p > 0.05$). However, soil VWC showed a marginally significant weak positive association with leaf RWC ($r = 0.31$, $p = 0.085$), consistent with higher VWC observed under VGDT compared with DT during the application period. In contrast, leaf RWC was strongly and positively correlated with large tuber number ($r = 0.61$, $p < 0.001$), and total stolon number was positively correlated with large tuber number ($r = 0.55$, $p = 0.001$). Maximum rooting depth was not significantly correlated with yield traits and was negatively associated with stolon number.

**Variable 1**	**Variable 2**	**Pearson r**	***p*-value**
Soil VWC	Leaf RWC	0.310	0.085
Soil VWC	Total stolons	–0.205	0.261
Soil VWC	Large tubers ≥ 9 cm	0.174	0.341
Soil VWC	Maximum rooting depth	0.204	0.264
Leaf RWC	Total stolons	0.216	0.236
Leaf RWC	Large tubers ≥ 9 cm	0.608	< 0.001
Leaf RWC	Maximum rooting depth	0.094	0.610
Total stolons	Large tubers ≥ 9 cm	0.545	0.001
Total stolons	Maximum rooting depth	–0.535	0.002
Maximum rooting depth	Large tubers ≥ 9 cm	–0.228	0.209
